# Simulated Partners and Collaborative Exercise (SPACE) to boost motivation for astronauts: study protocol

**DOI:** 10.1186/s40359-016-0165-9

**Published:** 2016-11-14

**Authors:** Deborah L. Feltz, Lori Ploutz-Snyder, Brian Winn, Norbert L. Kerr, James M. Pivarnik, Alison Ede, Christopher Hill, Stephen Samendinger, William Jeffery

**Affiliations:** 1 Department of Kinesiology, Michigan State University, East Lansing, MI 48824 USA; 2 Department of Psychology, Michigan State University, East Lansing, MI USA; 3 Department of Media and Information, Michigan State University, East Lansing, MI USA; 4 Universities Space Research Association, Houston, TX USA; 5 Department of Kinesiology, California State University, Long Beach, CA USA

**Keywords:** Behavioral health, Exercise, Exergames, Fitness, Köhler effect, Motivation, Relational agent, Software-generated partner, Virtual reality

## Abstract

**Background:**

Astronauts may have difficulty adhering to exercise regimens at vigorous intensity levels during long space missions. Vigorous exercise is important for aerobic and musculoskeletal health during space missions and afterwards. A key impediment to maintaining vigorous exercise is motivation. Finding ways to motivate astronauts to exercise at levels necessary to mitigate reductions in musculoskeletal health and aerobic capacity have not been explored. The focus of Simulated Partners and Collaborative Exercise (SPACE) is to use recently documented motivation gains in task groups to heighten the exercise experience for participants, similar in age and fitness to astronauts, for vigorous exercise over a 6-month exercise regimen. A secondary focus is to determine the most effective features in simulated exercise partners for enhancing enjoyment, self-efficacy, and social connectedness. The aims of the project are to (1) Create software-generated (SG) exercise partners and interface software with a cycle ergometer; (2) Pilot test design features of SG partners within a video exercise game (exergame), and (3) Test whether exercising with an SG partner over 24-week time period, compared to exercising alone, leads to greater work effort, aerobic capacity, muscle strength, exercise adherence, and enhanced psychological parameters.

**Methods/Design:**

This study was approved by the Institutional Review Board (IRB). Chronic exercisers, between the ages 30 and 62, were asked to exercise on a cycle ergometer 6 days per week for 24 weeks using a routine consisting of alternating between moderate-intensity continuous and high-intensity interval sessions. Participants were assigned to one of three conditions: no partner (control), always faster SG partner, or SG partner who was not always faster. Participants were told they could vary cycle ergometer output to increase or decrease intensity during the sessions. Mean change in cycle ergometer power (watts) from the initial continuous and 4 min. interval sessions was the primary dependent variable reflecting work effort. Measures of physiological, strength, and psychological parameters were also taken.

**Discussion:**

This paper describes the rationale, development, and methods of the SPACE exergame. We believe this will be a viable intervention that can be disseminated for astronaut use and adapted for use by other populations.

## Background

Astronauts need to adhere to high intensity exercise regimens to mitigate reductions in muscle strength and endurance, bone density, and reduced aerobic capacity that occur during long space missions. Exercise is also considered a key psychological countermeasure to risks of adverse behavioral health although much less is known about the dose-response relationship between exercise intensity and behavioral outcomes. While most astronauts are able to sustain high intensity exercise programs over a 4–6 month period (the average International Space Station mission duration), there is concern that for longer duration missions, such as Mars, a key impediment to maintaining intense exercise levels is motivation. Identifying motivational strategies and technologies to support high intensity exercise over long durations has not been explored. Exercise video games (exergames) have been marketed as a way to increase motivation and enjoyment of exercise by being entertaining, engaging and providing a means to interact with other players. Although many exergames involve competition among players, there has been little attempt to analyze what game features and interpersonal interactions would best motivate users to continue exercising with these games.

National Aeronautics and Space Administration’s (NASA) research is evaluating a new high intensity integrated resistance and aerobic training program (SPRINT) during 6 months of spaceflight on the International Space Station (ISS) (ongoing) and during 70 days of bed rest (which simulates a reduced gravity environment) [1]. Preliminary evidence from bed rest research studies suggests that a vigorous intensity exercise program during bed rest is very effective. However, bed rest studies involve SPRINT training for ~100 days during bed rest and the ISS study requires training for ~180 days. Eventual space exploration missions will require compliance with an exercise program for 2–3 years. Motivation and adherence to high intensity exercise, coupled with a socially isolating environment with atypical access to social support may compromise compliance, especially if astronauts’ regimens become monotonous. Exercise programs that enhance enjoyment, self-efficacy, and a sense of social connectedness may mitigate decrements in mood and feelings of social isolation [2, 3].

Group dynamics, using social psychological mechanisms such as social comparison and indispensability to group achievement, may be a useful means to address lack of motivation (i.e., the level of effort) for vigorous physical exercise [4]. Our research is designed to determine whether astronauts’ motivation to exercise at intense levels repeatedly over long durations can be improved using a virtual, software-generated (SG) partner--one that is anthropomorphic but clearly artificial and synthetic. People can respond socially to computers/software agents (also referred to as relational or social agents) and apply social rules much as if they were human. There is a strong research base suggesting humans can establish significant social relationships (e.g., keep promises and perceive virtual characters as teammates) with SG partners [5–8].

Traditional group or partner exercise leads to higher adherence than individualized exercise programs [9, 10], but structured group exercise is not possible for astronauts during space missions due to limited space and exercise equipment. In addition, exercising in pairs may be difficult to coordinate. Exercising with an SG partner offers several advantages (e.g., availability, flexibility, autonomy) over a live human partner. An SG partner’s abilities can be adjusted automatically over time to attain a level that may be the most motivating to the player, thereby hypothetically, keeping the player engaged and active. Additionally, exercising with an SG partner has the potential to make workout sessions more enjoyable, improve self-efficacy regarding physical performance capability and adherence to the regimen, and create a sense of social connectedness with the virtual character. The effects of an SG partner may be even stronger when used in socially isolated environments where there is little human interaction.

Active video games (i.e., exergames) have become increasingly popular and have been marketed as a fun way to increase people’s motivation to exercise [11]. Several studies have found that people are motivated to exercise with active games that are entertaining, engaging, and interactive [12]. However, even exergames can become boring within a short period of time if played in isolation [13]. Few exergames take advantage of the potential of group dynamics to motivate physically active play, and there has been little attempt to analyze what interpersonal interactions would best motivate people to use and continue exercising with these games [11].

Recent research has shown that an SG partner, who was moderately more capable than the participant in an exergame, increased the player’s physical activity persistence more than playing the game alone [6, 7]. This research is based on group motivation dynamics principles that stress upward social comparison and a sense of indispensability of one’s efforts to their more capable partner under conjunctive task conditions [14]. Under such conditions, the dyad team can persist no longer than its weaker partner--when the weaker member stops, it was impossible for the stronger partner to continue. Thus, motivation is likely to be enhanced when one sees his/her efforts as being highly instrumental in achieving team success [14]. However, to date, the motivating benefits of an SG partner have not been explored with physical exertion tasks over an extended period of time and at high exercise intensities required of astronauts. Further, whether the motivating benefits of exercising with an SG partner who is continually superior will attenuate over long-term intense exercise has not been explored. This gap in the literature is important because the full promise of an SG partner in exercise games for long-term exercise (e.g., simplicity of manipulating relative ability over time; and avoiding scheduling conflicts with a live partner) hinges on this question. Given previous research suggesting that humans will establish significant social relationships with SG partners [5, 15], we sought to explore related questions in the context of exercising with an SG partner over a period of 24 weeks.

### Specific aims and hypotheses

**Fig. 1:**
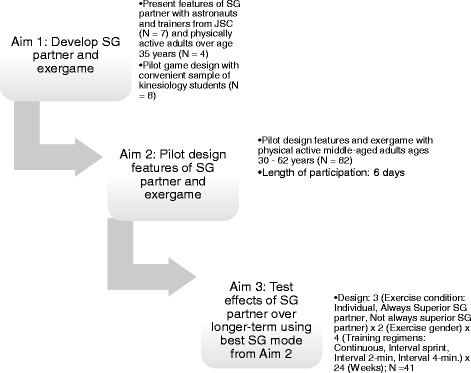
Overview of the aims and flowchart through phases of SPACE study

The primary aim of Simulated Partners and Collaborative Exercise (SPACE) was to determine whether recently documented motivation gains in task groups (dyads in particular) can be harnessed to improve motivation in an interactive exergame using SG partners. Aim 1 involved the development of software to create SG exercise partners and interface with cycle ergometers. The SG partner features were tested with focus groups of astronauts and NASA physical trainers and piloted with a convenience sample of physically active kinesiology students. After the SG exercise partners and games were developed, the design features of the SG partners, the exergame, and the questionnaires were pilot tested (Aim 2), using a short duration training study with master’s athletes and fitness club members who are similar in age and fitness to experienced astronauts. Aim 3 tested the long-term (i.e., 24 weeks) effectiveness of the SG partner in maintaining participants’ prescribed fitness goals, greater workout effort, physiological parameters (viz., aerobic capacity, ventilatory threshold, and musculoskeletal fitness), self-efficacy, enjoyment, interest in continuing the game, and perceptions of social connectedness compared to exercising alone (See Fig. 1 for an overview of the different aims and flowchart through phases). The following hypotheses were tested in Aim 3:

- H1: Exercising with a conjunctive SG partner over 24-week time period, compared to exercising alone, leads to greater workout effort and adherence.
- H2: Exercising with a conjunctive SG partner over 24-week time period, compared to exercising alone, leads to better aerobic capacity, higher ventilatory threshold, and greater thigh muscle strength.
- H3: Exercising with a conjunctive SG partner over 24-week time period, compared to exercising alone, leads to greater enjoyment in the activity, self-efficacy, interest in continuing the exergame, and sense of social connectedness.

## Method

### Study design

SPACE is a 6-month randomized control trial design. To better simulate actual astronauts, participants were middle-aged adults who are competitive athletes or highly physically active exercisers, recruited from mid-Michigan. Participants were assigned to one of three conditions: no partner individual control, an always superior SG partner, or an SG partner who was not always superior. Participants were asked to exercise on a cycle ergometer 6 days per week for 24 weeks using a routine consisting of alternating between days of moderate-intensity continuous (at or above 75% of their maximum heart rate) and three types of high-intensity interval sessions: (a) long, 4 × 4 min intervals at or above 90% HRmax with 3 min active rest (i.e., cycling at a recovery rate), (b) medium 6 × 2 min intervals at 70, 80, 90, 100, 90, 80% of HRmax, respectively with 2 min active rest, and (c) short, 30 s max sprint intervals with 20 s active rest. The design consists of a 3 (Exercise condition) × 2 (Participant gender) × 4 (Training regimens: Continuous, Interval sprint, Interval 2-min, Interval 4-min.) × 24 (Weeks) mixed design with repeated measures on the last two factors. Participants were able to vary cycle ergometer wattage to increase or decrease intensity only during the continuous and 4-min. interval sessions. We did not want the participants to overexert on the first half of the ladder on the 2-min intervals, and the sprint intervals were already set for maximum intensity. Table 1 contains details of the exercise regimen.

**Table 1:** Weekly exercise regimen for 24-week study

Day	Workout	Description
1	Continuous	5 min. warm-up starting at 50% of HRmax and progressing in intensity until Ss reach an effort that will elicit 75% max HR, followed by 30 min. of continuous cycling at or above 75% max HR. Ss are allowed to increase or decrease their work intensity.
2	Long intervals	5 min. warm-up starting at 50% of HR max. and progressing in intensity until Ss reach an effort that will elicit 90% max HR, followed by 4 × 4 min intervals at or above 90% max HR with 3 min. active rest (at 50% HR max). Ss are allowed to increase or decrease their work intensity.
3	Continuous	
4	Medium intervals	5 min. warm-upstarting at 50% of HR max. and progressing in intensity until Ss reach an effort that will elicit 70% max HR, followed by 6 × 2 min intervals at 70, 80, 90, 100, 90, 80% max HR with 2 min. active rest (at 50% HR max). Ss are not allowed to increase or decrease their work intensity.
5	Continuous	
6	Short, sprint intervals	After 10 min. warm-up, progressing in intensity until Ss reach an effort that will elicit max HR (100%), Ss pedal at that same workload for 8 × 30 s sprint intervals with 20 s active rest. Ss are not allowed to increase or decrease their work intensity.

Prior to conducting the intervention, we conducted focus groups of astronauts, astronaut trainers, and highly physically active middle-aged adults. In addition, pilot tests were carried out to refine the exergame and assessments. In the 6-month study, assessments were made on numerous physiological, performance, and psychological variables. This protocol paper adheres to the SPIRIT guidelines.

### Participants

#### Recruitment and eligibility criteria

There were three phases of recruitment: focus groups, piloting testing, and the 24-week intervention study. Each involved separate criteria.

#### Focus group recruitment

Recruitment of astronauts and astronaut trainers took place at the Johnson Space Center in Houston, TX. Eligibility for astronauts included long-duration flight and exercise experience and availability for interviews. Astronaut trainers at the Johnson Space Center were recruited based on availability in their schedules. No incentives were provided for participation. We prioritized for equal numbers of men and women.

A separate focus group of highly active middle-aged adults was recruited from the local community through the first author’s personal knowledge of local master’s athletes. Participants were required to be between 35 and 60 years of age and exercise at least four times per week at moderate to high intensity. One-half of the group was required to be female. No incentives were provided for participation. Two co-investigators (1 female), with focus group experience, conducted all focus group interviews.

#### Pilot testing recruitment

A convenience sample of university kinesiology majors, who were physically active and at least 18 years of age, were recruited to test the game and mechanics of the cycle ergometer-game interface for six sessions within a 2-week period. Flyers were posted throughout the kinesiology building and announcements were made in kinesiology classes. We attempted to recruit for equal numbers of men and women. Participants were told that they would be given a “Training Like an Astronaut” t-shirt for their participation. Testing was conducted by members of the research team.

Solicitation of participants for the 6-day pilot (Aim 2) was for those who were 30–62 years of age who would like to improve fitness and who exercise at least 30 min. per day, three times per week at moderate to high intensity. We strived for a mean age of 48 years for the sample, the mean age of an experienced astronaut. Participants were recruited from the local community through flyers posted at races, fitness centers, and athletic shops; through emails to running, triathlete, and cycling clubs; and through employee fitness programs. Compensation for the study included a “Training Like an Astronaut” t-shirt and $6.00 per session to cover mileage and parking to be received at the end of the study. Payment was not contingent on exercise performance.

Potential participants were screened using the Physical Activity Readiness Questionnaire (i.e., and excluded if they answered ‘yes’ to anyone of the following: have a heart condition that precludes participating in moderate to vigorous physical activity; feel chest pain during physical activity; feel chest pain while resting; lose balance because of dizziness, lose consciousness; have joint or bone problems that could be made worse by physical activity; if a doctor is currently prescribing medication for blood pressure or heart condition; or if there are any known reasons why the participant should not do physical activity). Also, all men over 44 years. had to obtain physician consent prior to participating.

Potential participants were also screened by self-reported physical activity levels. Eligibility required at least 30 min. of physical activity per day, three times per week at moderate to high intensity. Those who met the initial qualifications were given an incremental exercise test (cycle ergometer) in order to estimate their maximal oxygen consumption (VO_2_ max). Participants were required to reach an estimated VO_2_ max value of 35 ml/kg/min or achieve the 150-watt stage of the test. Any potential participants who did not meet the aforementioned requirements were excluded from the study. A total of six participants either self-selected out of the study after the incremental exercise test or did not qualify.

#### Twenty-four week intervention study recruitment

Solicitation of participants for the 24-week intervention study (Aim 3) was similar to the 6-day pilot study. However, recruitment has been conducted in two separate cohorts across 2 years because of space limitations to conduct the intervention. Compensation for the study included the same “Training Like an Astronaut” t-shirt and $6.95 per session. Participants received payment only for the number of days they complete for a potential total of $1000. Payment was provided on a monthly basis but was not contingent on performance. Instead of a graded exercise test, potential participants performed the same test as previously described for the six-day pilot study, but expired respiratory gases were collected during this test so VO_2_ was measured, rather than estimated. As was the case with the 6-day pilot study, participants had to reach at least 35 ml/kg/min or make it through 150 Watt stage of max test to qualify for this phase of the study. The astronaut average VO_2_ max is ~42 ml/kg/min. and we strived for that as a sample mean.

#### Study samples

The study sample for Aim 1 included two focus groups. The first group comprised four experienced astronauts (2 female) and three astronaut personal trainers (1 female). Participants reviewed the prototype design of the male and female SG partners and provided feedback on facial features and expressions, somatotype, voice, and verbal interactions. They also provided feedback on the features of the game, including the variety of exercise terrain, workout summary (average RPM, distance traveled, etc.), and the virtual trainer who provided game instructions. Based on feedback from the first focus group, a second focus group of four highly active male and female athletes/exercisers, over 35 years of age, reviewed a second version of the SG partner (more muscular, more expressive) and game (more varied terrain) that had been developed to further refine the appearance of the SG partners, exergame interface, and the nature and quality of interactions between participants and their SG partners (e.g., detail of introductions, greetings). After conducting focus groups, the game was pilot tested on a convenience sample of six highly active kinesiology students (2 female) at the university. They rode a stationary cycle on a simulated bike path for 30 min. for 6 days within 2 weeks to test game mechanics and protocol.

The study sample for Aim 2 consisted of 82 highly physically active adults, ages 30 – 62 years. These were participants who would be of similar age and fitness levels to those who would be recruited for the full intervention study.

For the long-term intervention (Aim 3), a total of 419 participants expressed interest in enrolling in the study. Of those, 221 completed a screening survey on Qualtrics. A final sample of 41 highly physically active adults, (18 female, 44%; one Hispanic) enrolled in the study in two cohorts. The first cohort of participants included 11 women, 12 men (*M* age = 46.74 ± 6.98). The second cohort included 7 women, 11 men (M age = 44.17 ± 9.31). We have strived for equal numbers of males and females, but in any case, insured proportional numbers of males and females in each condition.

Power analyses were performed using G*Power software (see gpower.hhu.de). To examine effects within and between treatment groups for the primary effort dependent variable, a repeated-measures ANOVA of three measurement blocks (for averaged exercise session data), with a moderate correlation among repeated measures (ρ = 0.3), suggested we would detect a moderate effect (f = .35) with the sample size of 13 individuals per group (total N = 39) with a probability of .95. These projections were consistent with previous conjunctive-partnered studies conducted by the research group that have shown large treatment effects (e.g., *d* = .99 [4]; *d* = 1.38 [16]).

### Description of SPACE exergame, cycle ergometer interface, and testing facility

The exergame, developed specifically for this study, incorporated the exercise regimen of continuous and interval training and included a series of different bike paths for each of the 6 days. SPACE includes a same-sex SG trainer who provides instructions for all of the workouts. In the partnered conditions, the game includes a same-sex SG partner who is introduced by the SG trainer as a teammate

Participants can view workout information on the screen, such as current intensity level, (measured in watts), RPMs, the distance cycled, whether they are above or below their target watts, and a clock that counts down from 30 min for the continuous workout protocol or for the specific interval time they are working on for interval days. Participants can change intensity or bike speed (+/- 5, 10, or 20 watts) by selecting the appropriate buttons on their keypad. Because of the design of the cycle ergometer, participants cannot change their intensity by pedaling faster or slower —for example, pedaling more slowly results in an increase in resistance, keeping the overall intensity constant.

SPACE is interfaced with the Monark LC4 cycle ergometer (with adjustable seat and handle bar) and is used in conjunction with a PC, monitor, and numeric keypad. Participants pedal the cycle ergometer at a fixed wattage based on a prescribed percentage of their target HR_max_ while viewing their gameplay via the computer monitor. The testing facility has six exercise cubicles and can accommodate up to six participants at a time. There is a separate room to perform pre- and post-session measures (e.g., blood pressure). Additionally, another lab in the building houses all physiological equipment relevant to the current investigation, including an isokinetic machine (Biodex 3 dynamometer), body composition (Bodpod), and metabolic carts (Parvo).

### Intervention

All qualified participants had 6 days of baseline cycling in Week 1, using the 6-day exercise regimen, to adjust their work intensity (as set in watts) without the SPACE game. In Week 2, all participants were introduced to the SPACE game, known to them as “Training Like and Astronaut” and went through the no-partner Control condition of the 6-day exercise regimen at their target watts intensity, where an SG same-sex trainer appeared on the monitor and provided instructions for the workouts. After Week 2, the project manager randomized participants, balanced for gender, to one of three conditions: no-partner Control, Always superior partner (AWS), or Not always superior partner (NAS). Participants were blinded to the conditions they were in and were told not to discuss their exercise with anyone who might be in the study. Although experimenters were not blind to conditions, they were unaware of the experimental hypotheses.

#### Control

In the individual control condition, participants cycled under the same conditions and instructions as in the previous week. They must try to cycle at their target watts or higher for the Continuous 30-min. and 4-min intervals sessions; however, if they feel they cannot cycle at that intensity, they can lower their watts. They received feedback, prior to each session, on their previous workout performance for the type of workout they have that day (i.e., continuous 30-min, 4-min interval, etc.).

#### Always superior partner (AWS)

Participants were told that they will be cycling for the rest of the study with an SG partner who will be their teammate. They were told that their teammate, named Chris (always same sex as participant), is programmed to be slightly more fit than they are, but that he/she is designed to respond to a workout as any person would, and can experience fatigue at some point during the exercise session. This was manifested in two ways: (a) false feedback on potential initial, baseline performance from Week 2 (the SG partner was alleged to have been programmed to cycle about 1.15 times faster than the participant) and (b) during the exercise session, the image of the SG partner was shown on the video monitor starting out at the same pace as the participant but quickly moving into a faster pace and then always be shown to be outperforming the participant. The 15% discrepancy between participant and SG partner was determined from feedback in focus-group testing.

The SG trainer explained the nature of the conjunctive task (i.e., that they are working together toward a team workout score, which is determined by whoever bikes the shorter distance). Further, they were told that they and their teammate are linked together, so that if one of them cycles too far ahead AND the other is below their target watts, the team member who is ahead will be slowed down, until the gap has lessened. When this happens, they will also see a red hue on the bottom of the screen, indicating that their teammate has had to slow down.

In order to build rapport with the SG partner, participants were introduced to him/her, through a guided dialogue-tree interface which allows participants to respond to questions posed by their SG partner by selecting from several pre-programmed responses on screen. Examples include asking if the participant is from Michigan with response choices of “Yeah, I’m a Michigander” or “Actually, I’m not from around here.” Depending on the choice option chosen, the SG partner responds with appropriate follow-up dialogue and additional questions. There are five question and response interactions. The type of personal information exchanged was developed through focus group testing as part of the SG development the SG partner.

In terms of continued rapport-building, the SG partner comments periodically throughout the 24-week intervention, either before or after the workout. This occurs a total of 32 times with comments such as, “Ready”; “Good work”; and “I’m looking forward to our workout.” In addition to the introductory dialogue, there are three other occasions where a dialogue-tree format occurs: (a) in Week 13 at the halfway point of the study, (b) in Week 21 with 1 month to go, and (c) on the last day, where the SG partner thanks the participant for working out with him/her and says goodbye and the participant is then given the option to reply.

As a part of designing the SG partner to have human qualities but still be recognized as a computer, there are two separate weeks when he/she is not available. In Week 9, the SG partner is sick with a software “virus” for 4 days, and in Week 19, he/she has an injury and is out for the week.

Just before participants begin the game after meeting their SG partner for the first time, the SG trainer explains that they can see their average watts and the distance they biked in their first session (Week 2 baseline) compared with their SG partner’s average watts and distance. The SG partner’s performance also shows a 15% better score. They are then reminded that their workout score for this session is determined by whoever cycles the shorter distance in 30 min. continuous workout (or 4-min interval) and, that on the screen, they will be able to see both their own score and the team score. They were instructed that if they finished ahead of the SG partner, the partner’s score would be the team score. If the SG partner finished ahead of them, their own score would be the team score. At any time that the participant is ahead of the SG partner, the partner is visible in a profile view in the corner of the screen. As in the Control condition, participants receive feedback, prior to each session, on their own previous workout performance for the type of workout they have that day.

#### Not always superior partner (NAS)

All information provided to the NAS participants is the same as that provided to those in the AWS condition. The only difference in the two conditions is that the participant was sometimes be able to surpass the SG partner. This happened on 17 of the possible 117 occasions (15%) where participants cycled with a partner. The 15% was a best guess on how to both maintain the impression of partner superiority and minimize participant discouragement based on focus group responses. There are eight times during continuous sessions and three times during each of the interval sessions. During these occasions, the NAS partner says things like, “Sorry, I just couldn’t keep up today” or “Those sprints were tough!”.

### Procedures

Prior to the start of the study, participants were invited to an information and orientation session in the facility where the study would be conducted. The session provided information on the pretest measures that would be conducted (VO_2_ max test, muscular strength test of quadriceps and hamstrings, body composition), compensation for participation, and the schedule. Participants could choose either a Sunday through Friday or a Monday through Saturday time schedule. Participants were then scheduled for their pretest assessments. Descriptions of these pretest measures are detailed under Measures.

#### Pretest assessments

Pretest assessments were scheduled 1 to 3 weeks prior to the first week of the study. Participants came to the exercise testing lab and were first administered the informed consent to participate in the study. If participants consented, they then were given the instructions and were evaluated for body composition and performed the VO_2_ max test. After the completion of the VO_2_ max test, researchers indicated whether participants met the eligibility criteria for VO_2_ max. If participants met criteria, they were scheduled for a second lab visit where their lower body muscular strength was measured. After the second testing visit was completed participants were then ready to begin participating with the exergame.

#### Week 1

During the first week of the study, participants came to the exercise facility to get accustomed to the 6-day exercise regimen, outlined in Table 1. Exercise was initially prescribed based on percentages of VO_2_max and was monitored by HR. For example, the HR and workload corresponding to 90% of VO_2_max during the initial max test was recorded as the training target for the 4 × 4 min intervals. Participants performed all of the workouts under the supervision of research staff with an exercise physiology background and the research staff adjusted the participant’s work intensity if necessary based on the HR. If adjustments to participants’ cycle power output were made by the research staff, the adjusted watts were used for all subsequent sessions. Participants were not exposed to the game in this adjustment week. Prior to the start of each session (during this week and throughout the entire study), participants were fitted with a Polar HR monitor and a blood pressure cuff for pre-exercise cardiovascular measurements.

#### Week 2: baseline

As described previously, during Week 2, all participants cycled in the Control condition, which includes having an SG trainer to provide instructions. Participants were instructed to wear the headphones provided or to bring their own so that they could hear the instructions and not overhear participants in adjacent exercise stations. HR, blood pressure, and rating of perceived exertion (RPE) measures were taken during each session of baseline and continued throughout the study. During the continuous workout, HR was collected at 10, 20, and 30 min. RPE was collected at the end of the workout (30 min. mark). HR and RPE were taken at the end of each 4 × 4 min. interval (4, 11, 17, and 25 min.). During the sprint workout, RPE was collected at the end of the 10 min. warm up and at the end of the 8^th^ sprint. HR was collected at the end of the warm-up and after each of the 8 intervals in the sprint workout. HR was collected at the end of each of the 6 × 2 min. intervals and RPE was collected at the end of the 4^th^ and 6^th^ intervals. At the end of the workouts, participants were required to cool-down for 3 min. or until HR was below 130 beats per min. Blood pressure was taken after the workout and the participant was given a survey if there was one for the day they just completed.

#### Week 3 through 24

Starting in Week 3, participants were randomly assigned to one of the three conditions. All procedures remained the same. During Week 13, participants completed midpoint VO_2_ max, body composition, and leg strength testing using the same protocol from the beginning of the study. Using the fitness data from the VO_2_ max test, adjustments could be made to prescribed watts for participants if training effects had occurred. If participants improved their VO_2_ max, prescribed watts were adjusted to the new regression line that predicts their performance at a variety of intensities.

Strategies to improve adherence to the intervention included follow-up phone calls if participants missed a session to try to minimize any drop out or adherence problems. Occasional nonparticipation was anticipated as inevitable and managed afterward in missing data analysis. The project manager also established a secure and direct online communication with all participants to encourage proactive notification and management of any potential issues with scheduled session appointments. This deterred missed appointments that occur due to multiple inherent, non-study personal schedule conflicts. Adherence to the protocol was monitored in the lab by staff. Participation would be discontinued for any individual who incurred an injury, within or outside of the exercise sessions that could be aggravated by continuing the exercise program.

### Measures

Mean change in cycle ergometer workload (Watts) from the initial continuous and 4 min. interval session is the primary dependent variable reflecting motivational effort. Measures of physiological, strength, and psychological parameters (perceived effort, enjoyment, self-efficacy, and social connectedness) were also obtained. A schedule of the frequency of measures administered is contained in Table 2.

**Table 2:** Frequency of measures

Measure	Applicable workouts	Frequency
Fitness variables: VO2 max Ventilatory threshold Thigh strength Body composition	Not specific to a workout	Weeks 1, 12, and 24
Free-living physical activity	Not specific to a workout	Weekly
Rating of perceived exertion	Continuous Sprint intervals Short intervals Long intervals	Daily during sessions
Heart rate	Continuous Sprint intervals Short intervals Long intervals	Daily during sessions
Blood pressure	Not specific to a workout	Before and after each session
Self-efficacy	Continuous Long intervals	Weeks 2, 4, 8, 12, 17, 20, 24
Enjoyment	Continuous Sprint intervals Short intervals Long intervals	Weeks 1, 2, 3, 9, 24
Social connectedness	Not specific to a workout	Weeks 12, 24
Team perceptions	Not specific to a workout	Weeks 3, 12, 24
Alternative Godspeed Indices	Not specific to a workout	Weeks 3, 12, 24
Game interest	Not specific to a workout	Week 24

#### Primary measures

##### Effort

Mean change in cycle ergometer workload (watts) from each participant’s initial targeted workload (individually determined during max testing), as well as mean change in workload over 24 weeks from baseline are the primary dependent variables reflecting work effort for both the continuous and 4-min. interval sessions. Without missing data, there are a total of 66 Continuous, 22 4 × 4 min interval, 22 6 × 2 min interval, and 22 sprint interval effort measures. Effort to persist in the 2-min interval workouts was measured in the number of seconds completed without decreasing intensity. Effort to persist was also measured in the sprint workouts by the number of intervals completed (up to a maximum of 8). Participant-controlled changes in watts above the target were not allowed in the 2-min interval workout, though they could decrease their watts if the workout at their prescribed watts was too difficult. In the sprint interval, participants could not decrease watts, but they could stop if unable to continue.

##### Rating of perceived exertion (RPE)

Along with an objective measure of effort, participants reported their subjective evaluation of effort levels throughout exercise, using the 15-point version of the Borg RPE scale (minimal effort = 6; maximum effort = 20, which when multiplied by 10 corresponds to an estimate of heart rate). Ratings were then averaged for each experimental session. We assessed RPE because perceptions of exertion also may influence one’s motivation to persist at a taxing task [17].

##### VO_2_max. and ventilatory threshold

The test was conducted using an electronic cycle ergometer, and expired respiratory gases were collected using a Parvo metabolic cart. The test ended when participants reached volitional exhaustion and stopped pedaling or were instructed to stop when they reached two out of three criteria indicating that they had achieved VO_2_max (i.e., plateau in VO_2_ occurred, heart rate was higher than 95% of predicted, and respiratory exchange rate was over 1.05.) Ventilatory threshold values were calculated from the VO_2_max test data by identifying the breakpoint of pulmonary ventilation (VE) from VO_2_ as described by Amann et al. These tests were measured at the time of screening, at Week 13, and at posttest [18].

##### Quadriceps and hamstring strength

Isometric muscular strength was measured at the right knee (45 deg, 5 s contraction) including peak torque extension and peak torque flexion using the Biodex 3 dynamometer. Participants warmed-up with four repetitions of extension and flexion, followed by 3 repetitions each for the actual test. These tests were performed at the time of screening, at Week 12, and at posttest.

##### Body composition

Each participant’s body density was measured via air displacement plethysmography (BodPod), and %fat and fat free mass were calculated according to standard equations.

#### Secondary measures

A number of psychological measures were taken to assess participants’ enjoyment, self-efficacy, social connectedness, and interest in the game. In addition, measures of participants’ perceptions of the SG partner were assessed for those in the partnered conditions.

##### Enjoyment, social connectedness, and interest in the game

Enjoyment was assessed with a 5-item version of the Physical Activity Enjoyment Scale [19] regarding “how you feel at the moment about the physical activity you have been doing.” The Social Connectedness Scale [20] was adapted to include six items to measure participants’ sense, in general, of companionship, togetherness, and relatedness during the exercise sessions. Game interest was assessed on the final day with a single item that asked participants to rate their interest in playing an exercise video.

##### Self-efficacy

Self-efficacy beliefs were measured pre- and post session for the continuous and 4-min interval workouts only. Pre-session efficacy was rated in terms of the up-coming workout and post-session efficacy was rated regarding beliefs about the next time the participant engaged in that workout. Participants rated their confidence on an 11-point probability scale that they could cycle for 30 min (continuous workout) at six different intensities, starting at 75% of the participant’s VO_2_max and increasing by 5% for each item up to 100%. For the 4-min interval workout, participants rated their confidence on the same scale that they could complete all four, 4-min intervals (4-min interval workout) starting at 90% of their VO_2_ max and increasing by 5% up to 100% (i.e., 3 items).

##### Additional perceptual measures

To check for perceptions of the SG partner’s humanness, participants in partnered conditions completed a questionnaire that used the Alternative Godspeed Indices [21]. To check for participants’ perceptions of their partner as a teammate, partnered participants completed a 5-item questionnaire that assessed their perceptions of the working relationship with their partner (e.g., I felt I was part of a team) [22, 23].

##### Monitored measures

Blood pressure and HR were monitored for safety. Blood pressure was measured using a GE Dinamap automatic blood pressure monitor. HR was measured using the Polar HR monitor during each exercise session. Additionally, participants were asked to recall any outside activity and record the activity and the duration they participated in the activity to account for any outside influences on performance and fitness measures.

### Statistical analyses

For the continuous and 4-min. sessions, a blocked session repeated-measures analyses of variance (ANOVA) will be utilized to examine the effect of treatment on the primary motivational effort measure (mean change in power output) across conditions and across the study. Group and individual linear trend analyses and growth curve modeling will be applied to assess potential inter- versus intra-individual variability trajectories and patterns of change over the 24 weeks. ANOVA will be used to analyze the mean differences for percent of cycling 2-min. intervals and 30-s. sprints at or above targeted level of effort. In all analyses of these measures, baseline performance measures will also be covaried to control for pre-program levels of fitness. Missing data will be evaluated for randomness and a within-subjects, same-session type linear interpolation will be used to impute the data set. Interpolation of missing data was chosen due to the nature of the effort measure (i.e., longitudinal objective measure of exercise effort).

Hypotheses related to the effect of treatment on objective measures of fitness (e.g., VO_2_max measures; ventilatory threshold; isokinetic strength) will be tested using ANOVA at the pre, mid, and post-study time points.

Given that prior research has found little evidence that enhanced effort achieved with conjunctive exercise partners is accompanied by any aversion to the exercise task, loss in self-efficacy, or rise in subjectively experienced exertion, we will check to see if these patterns replicate in this study. We will also examine self-reports of social connectedness both generally and toward the SG partner, in terms of feeling like a teammate. For each variable, we will use a general linear model to analyze means across the multiple time points administered and for the entire study, as well as correlations to blocked means for effort measures. We also will check for sex and age differences.

## Discussion

This paper describes the rationale, development, and methods of the SPACE exergame program. This intervention was designed to maintain intense levels of exercise for astronauts and other adults who need to exercise at vigorous levels for health and performance purposes. We believe this will be a viable sustainable intervention that can be disseminated for astronaut use and adapted by other populations. The strengths of the study include the strong conceptual basis of group dynamics, using social psychological mechanisms such as social comparison and indispensability to group achievement to boost motivation (i.e., the level of effort) for vigorous physical exercise [4]. In addition, the use of an SG partner embedded in an exergame has several practical advantages over a live human partner for astronauts as well as other populations. For instance, an SG partner’s abilities can be adjusted automatically over time to perform at a level that is always challenging to the player, thereby keeping the player engaged and active.

The study also has some limitations. The exercise program uses only the aerobic segment of NASA’s SPRINT exercise regimen. Including the weight training component would provide a more thorough test of the effectiveness of an SG partner to sustain motivation in a vigorous exercise program. Additionally, participants did not live in an isolated environment, similar to what astronauts experience at the ISS or on deep space missions. The effects of an SG partner may be even stronger when used in an environment where inhabitants are socially isolated, especially in testing its social connections effects. Further, the interactions with the SG partner were relatively superficial and limited by the software. Having an SG partner with some artificial intelligence capabilities could enrich the social interaction such that the SG partner is more than just a cycling partner but also a relational agent.

The authors plan to communicate results to participants and through a local community presentation. We plan to communicate scientific results through presentations at professional conferences and through publications in scientific journals.

## Abbreviations

AWS: Always superior condition
HR_max_: Maximum heart rate
IRB: Institutional Review Board
ISS: International Space Station
ml/kg/min: Milliliters per kilogram per minute
NAS: Not always superior condition
NASA: National Aeronautics and Space Administration
NSBRI: National Space Biomedical Research Institute
PAES: Physical Activity Enjoyment Scale
RPE: Rating of perceived exertion
SG: Software-generated
SPACE: Simulated Partners and Collaborative Exercise
SPRINT: NASA’s high intensity integrated resistance and aerobic training program
VE: Volume of expired respiratory gases
VO_2_ max: Maximum rate of oxygen consumption
